# Socio-cognitive mindfulness in nursing: A scoping review

**DOI:** 10.1371/journal.pone.0300459

**Published:** 2024-04-29

**Authors:** Mikyoung Lee, Hyunyoung Park

**Affiliations:** 1 Department of Nursing, Dongshin University, Naju-si, Jeollanamdo, South Korea; 2 College of Nursing, Chonnam National University, Gwangju, South Korea; Alzahra University, ISLAMIC REPUBLIC OF IRAN

## Abstract

**Background:**

While research on meditative mindfulness in nursing is abundant, research on socio-cognitive mindfulness is in its early stages despite its potential advantages to nursing practice and nursing education. This study introduces the under-examined concept of socio-cognitive mindfulness to the nursing field.

**Objective:**

To identify what is known in the research field of socio-cognitive mindfulness in nursing. Specific aims were to identify the effects of socio-cognitive mindfulness on nurses and nursing students, and the application of socio-cognitive mindfulness interventions and their effectiveness in nursing.

**Design:**

A scoping review following the Arksey and O’Malley framework.

**Methods:**

An electronic search of PubMed, EMBASE, Cochrane Library, CINAHL, Web of Science, ERIC, and ProQuest databases was conducted. The search included full-text papers published in peer-reviewed journals in English. The included studies were independently examined by the two authors to ensure the thorough inclusion of relevant research by screening of titles and abstracts and screening of full-texts. The selected papers were categorized based on the specific objectives of the review.

**Results:**

Out of 5,798 papers, six quantitative studies and one mixed method study were included in the review. Among the seven studies, two studies investigated the effects of socio-cognitive mindfulness on nurses, four examined the effects of socio-cognitive mindfulness on nursing students, and one conducted an intervention study applying socio-cognitive mindfulness and identified its effects on nurses. The findings revealed several benefits of applying socio-cognitive mindfulness to nursing practice and nursing education. Specifically, socio-cognitive mindfulness enhanced nurses’ and nursing students’ positive emotions and effective emotion regulation, which would positively influence nurses’ nursing performance as well as students’ academic outcomes and quality of college life.

**Conclusions:**

This study raises researchers’ awareness of the significance of socio-cognitive mindfulness in nursing, and strongly recommends applying socio-cognitive mindfulness to nursing practice and nursing education and evaluating its effects.

## Introduction

Mindfulness is to pay attention to the present moment and observe the present experiences with wakefulness [1]. Mindfulness is a well-known construct as an effective strategy for emotion regulation [2]. That is, it is critical for individuals to possess mindful attitudes in order to manage their emotions effectively. Compared to students of other majors and professionals in other fields, mindful attitudes are much more helpful to nursing students and nurses, who need more effective strategies to manage their emotions, considering the situations they are placed in [3, 4]. For example, nursing students experience more stress and unpleasant emotions due to the intense amount of study required and their clinical practicum [5]; nurses confront unfavorable work situations that require effective emotion regulation strategies for their psychological health and ultimately for better nursing practice [6].

There have been two leading schools of thought on mindfulness: one is meditative mindfulness developed by Kabat-Zinn [7], and the other is socio-cognitive mindfulness pioneered by Langer [8]. Kabat-Zinn’s meditative mindfulness is based on Eastern philosophy and focuses on non-judgmentally paying attention to the present through meditation, while Langer’s socio-cognitive mindfulness underlines the cognitive tendencies and attitudes of Western perspectives. Socio-cognitive mindfulness, unlike meditative mindfulness which is familiar to most researchers, argues that short-term interventions can have the effect of mindfulness by applying a variety of simple methods without a long period of meditation or training [9]. In particular, socio-cognitive mindfulness emphasizes flexibility with the environment by promoting openness to external stimuli through simple cognitive interventions without meditation [10, 11]. For example, in problem-solving situations, individuals can try to consider the situation in various different contexts and discuss possible solutions openly with others.

A plethora of research on meditative mindfulness has demonstrated a variety of positive effects in many fields, including the psychology, medicine, education, and nursing domains. In particular, Kabat-Zinn [12] implemented the mindfulness-based stress reduction (MBSR) program based on meditative mindfulness to reduce clients’ psychological symptoms, such as depression and anxiety, as well as patients’ physical pain [10, 13]. Recent studies related to COVID-19 reported the mediating role of mindfulness between self-efficacy and fear of COVID-19 [14] as well as between hardiness and stress of COVID-19 in university students [15]. In addition, mindfulness-based compassion therapy improved sleep quality and life satisfaction in elderly women [16].

Regarding nursing research, mindfulness-based programs for nurses alleviated their negative psychological symptoms, such as burnout, stress, mental distress, depression, and anxiety [17–19]. Mindfulness-based programs also enhanced nurses’ life satisfaction, empathy, emotional experiences, vocation, resilience, and coping abilities, which proved to be effective in not only personal life but also work and interpersonal relationships [18, 20]. For nursing students, meditative mindfulness helped them reduce stress and anxiety, while it improved their stress management skills, coping abilities, and empathy [21–23]. Through meditative mindfulness, nursing students were able to augment their self-esteem, happiness, and psychological well-being and, at the same time, alleviate study stress and depression [24, 25]. As described above, in countless studies meditative mindfulness has been reported as an effective means of improving individuals’ psychological well-being and physical health.

In comparison, research on socio-cognitive mindfulness has not flourished as much as research on meditative mindfulness; some studies exist in psychology, medicine, and education fields. In the psychology discipline, researchers have found that socio-cognitive mindfulness has been shown to be linked to psychological well-being and physical health as well as to cognitive performance. Socio-cognitive mindfulness, for instance, improved the immune system and life satisfaction, and reduced anxiety and depression by inducing cognitive flexibility [10, 11]. It also improved self-acceptance [26] and satisfaction in interpersonal relationships [27], while alleviating burnout and stress [28]. In the medicine field, simple interventions of socio-cognitive mindfulness effectively reduced psychological distress, such as depression and negative moods [29]. Socio-cognitive mindfulness also inhibited maladaptive emotion regulation strategies like rumination in both healthy individuals and clinical patients [29]. In the educational contexts, Langer [9] found that students could improve creativity and learning skills, such as cognitive performance, attention, memory, concentration, and problem-solving skills through mindful thinking. Additionally, students’ socio-cognitive mindfulness correlated positively with a desirable emotion regulation strategy (i.e., reappraisal) and positive emotions, but it correlated negatively with negative emotions [30]. Students’ socio-cognitive mindfulness was negatively associated with negative symptoms, such as compulsive tendency, depression, and anxiety; however, it was positively associated with quality of life [31]. Nevertheless, nursing researchers have yet to pay much attention to socio-cognitive mindfulness.

Socio-cognitive mindfulness is the process of creating new categories of information in open and creative ways, and it looks at phenomena from multiple perspectives by taking contextual situations into consideration [26, 32, 33]. Socio-cognitive mindfulness might be particularly beneficial for nursing students and nurses, aligning with the dynamic demands of their practice. Emphasizing heightened consciousness, flexibility, and engagement [10, 11], this approach would foster decision making and creative problem-solving, essential skills in the nursing profession. In addition, socio-cognitive mindfulness offers immediate benefits through short-term interventions [9], and it effectively reduces stress and negative emotions while promoting positive well-being [30]. This active and adaptable nature makes it suitable for busy nursing settings and education, eliminating the need for prolonged meditation training [10, 11].

Thus, we believe that there are potential advantages in applying socio-cognitive mindfulness to the context of nursing practice and nursing education. Considering little research on socio-cognitive mindfulness in nursing, we introduce this under-examined construct socio-cognitive mindfulness to the nursing field using a scoping review and suggest applying socio-cognitive mindfulness to the field. Specifically, this review aimed to identify what is known in the research field of socio-cognitive mindfulness in nursing.

## Methods

This review adopted the Arksey and O’Malley [34] framework for conducting scoping reviews. This framework consists of six stages: 1) identifying research questions, 2) identifying relevant studies, 3) study selection, 4) charting the data, 5) collating, summarizing, and reporting the results, and 6) consultation (this is an optional stage and is not implemented in this review). In addition, this scoping review followed the Preferred Reporting Items for Systematic Reviews and Meta-Analyses (PRISMA) statement for reporting scoping reviews [35].

### Stage 1: Identifying research questions

To formulate the research questions, the Population (Participant), Concept, and Context (PCC) framework described by the Joanna Briggs Institute [36] was used. According to the PCC framework, the population, concept and context in this study include nurses and nursing students, the effects of socio-cognitive mindfulness, and nursing practice and nursing education, respectively. Specific research questions are as follows:

Research question 1: What are the effects of socio-cognitive mindfulness on nurses?
Research question 2: What are the effects of socio-cognitive mindfulness on nursing students?
Research question 3: What are the interventions applying socio-cognitive mindfulness and their effectiveness in nursing practice and nursing education?

### Stage 2: Identifying relevant studies

The search was conducted with the help of a librarian specializing in health sciences. An electronic search was performed on the databases PubMed, EMBASE, Cochrane Library, CINAHL, Web of Science, ERIC, and ProQuest on February 9, 2023. The databases were chosen based on the Bidwell and Jensen’s COSI (COre, Standard, Ideal) model [37]: PubMed, EMBASE, and Cochrane Library were selected for the Core search in healthcare research; CINAHL and ERIC were selected for the Standard search in nursing research; Web of Science and ProQuest were selected for the Ideal search.

The search incorporated a wide range of sources, including not only peer-reviewed studies but also grey literature like dissertations. Given that the concept of socio-cognitive mindfulness is under-examined in nursing research, grey literature was included to ensure a comprehensive search. Studies published in English with full-text available were included. No restrictions on publication years were imposed to capture a comprehensive selection of relevant studies. Finally, the reference lists of the included studies were manually examined to ensure the thorough inclusion of relevant research.

A comprehensive search strategy was formulated by combining key terms using free text terms and MeSH terms for socio-cognitive AND (nurses OR nursing students). To expand and refine the search, Boolean operators (AND/OR) and asterisks at the end of keywords were utilized. The S1 File presents details of the search strings. For example, the following search terms were used for PubMed: ((((nurse[MeSH Terms]) OR (nursing[MeSH Terms])) OR (Students, Nursing[MeSH Terms])) OR (nurs*[Title/Abstract])) AND ((mindfulness[MeSH Terms]) OR (mindfulness[Title/Abstract])).

### Stage 3: Study selection

The process of study selection occurred through two phases: 1) screening of titles and abstracts and 2) screening of full-texts. The two authors (ML, HP) independently participated in the initial screening of titles and abstracts. Any discrepancies were addressed through discussion. In instances where consensus could not be reached, a third-party researcher (nursing professor) whose research interest included mindfulness intervened to make a final decision.

#### Inclusion and exclusion criteria

We considered both quantitative and qualitative studies. We included full-text articles published in peer-reviewed journals and studies that fulfilled the criteria of our study design. We excluded the following types of papers: papers whose full texts were not available, not original research papers (e.g., protocol, reviews), papers that did not focus on socio-cognitive mindfulness (e.g., meditative mindfulness, mindfulness-based intervention/practice/program/training/workshop, mindfulness-based stress reduction, cognitive behavioral therapy, and spiritual related ones), papers that did not identify the effects of socio-cognitive mindfulness, and papers not published in English.

### Stage 4: Charting the data

After identifying the eligible studies, the two authors (ML, HP) independently evaluated the full texts. Relevant data were then manually extracted from the chosen articles using a standardized data extraction form developed by the authors in Microsoft Excel for this specific purpose. The extracted data were as follows: the first author, publication year, country, study aims, study design, population and setting, the instrument to measure socio-cognitive mindfulness, any interventions delivered (if applicable), key findings including estimates (such as ‘r’, ‘β’ and/or mediation) and their significances for relevant variables, and conclusions.

### Stage 5: Collating, summarizing, and reporting the results

The included studies were categorized based on the three specific objectives of the review. The extracted data were synthesized, summarized in a tabular form, and presented in a narrative summary, considering the objectives that directed the scoping review.

## Results

**Fig 1:**
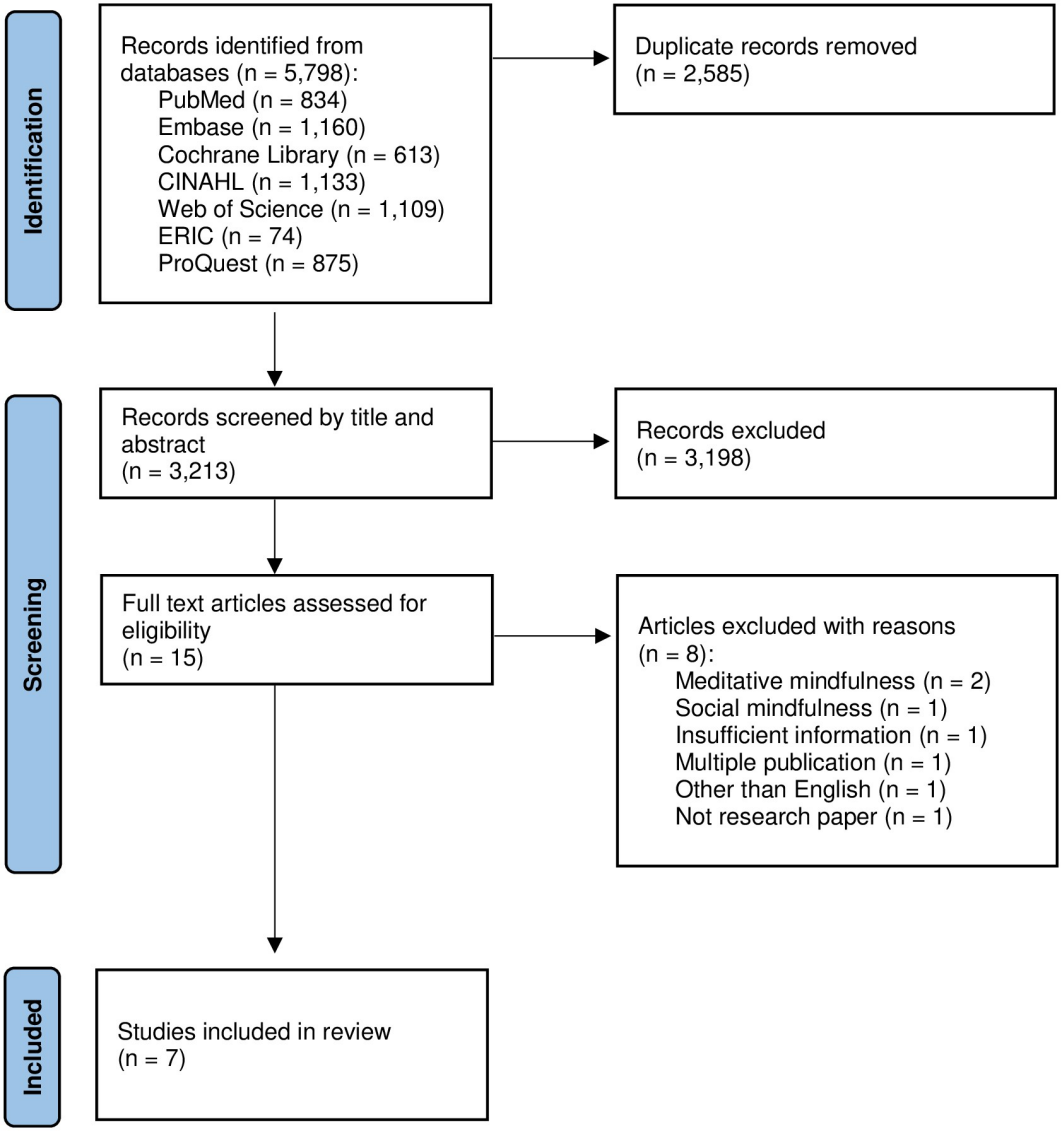
PRISMA flow diagram for included articles.

Fig 1 displays the study selection process using the PRISMA flow diagram, outlining the inclusion and exclusion of papers. Initially, 5,798 articles were identified via electronic database searches. After removing duplicates, 3,213 records underwent inclusion screening. Among these, 3,198 articles were excluded based on title and abstract assessment, leaving 15 articles for eligibility assessment. Subsequently, 8 articles were excluded after a full-text examination for the following reasons: research on meditative mindfulness (n = 2), research on social mindfulness (n = 1), research mentioning Langer’s socio-cognitive mindfulness but not containing any related contents (n = 1), research not identifying the effects of socio-cognitive mindfulness (n = 1), multiple publication (n = 1), research published in Norwegian (n = 1), and no research paper (n = 1). In particular, the reason for excluding one study on social mindfulness [38] was because social mindfulness is a different concept from the socio-cognitive mindfulness investigated in our study. Social mindfulness is defined as a motivational mindset in which individuals, during interpersonal interactions, take into account the needs and interests of others, aiming to enhance others’ control over their own outcomes [39]. Finally, 7 papers were included in the review. Among the 7 papers, 2 publications used the same sample [40, 41]; we treated them as separate independent studies since their research purposes were different.

### Characteristics of the studies

Table 1 summarizes the characteristics of the seven included studies. The studies were conducted between 2010 and 2022, with all the studies conducted after 2017 except one [42]. They consisted of five journal articles [40, 41, 43–45], one doctoral dissertation [42], and one master’s thesis [46]. Geographically, two studies were conducted in the United States [42, 46], four studies were conducted in South Korea [40, 41, 43, 44], and one study was conducted in Sweden and Norway [45]. Six studies used quantitative research designs with a validated instrument of Langer Mindfulness Scale (LMS) [47] to measure participants’ socio-cognitive mindfulness [40–45]. The one remaining study employed a mixed method [46]. The study populations were comprised of nurses and nursing students, with the sample size ranging from 156 to 459 in six quantitative studies and 22 nurses in the mixed method study.

**Table 1:** Characteristics of the included studies.

Author & Year (Country)	Journal	Study Aims	Study Design	Population	Instrument to Measure Socio-cognitive Mindfulness (Cronbach’s α)	Results (r and/or β and/or mediation)	Conclusions
Heard, 2010 (USA)	Unpublished doctoral dissertation	To determine the effects of mindfulness on comfort, work satisfaction, and burnout in nurses	A quantitative design with a questionnaire	186 nurses from four hospitals	Langer Mindfulness Scale (α = .87)	Socio-cognitive mindfulness and burnout: emotional exhaustion (r = −.209, p = .01), depersonalization (r = −.174, p = .05). Socio-cognitive mindfulness to emotional exhaustion (β = −.199, p = .003), to depersonalization (β = −.089, p = .021), and to personal accomplishment (β = .188, p < .001)	More mindful nurses would have fewer problems with the experience of depersonalization and emotional exhaustion. These nurses would seem to experience higher levels of personal accomplishment, thus possibly making them less prone to experience burnout in their work.
Sundling et al., 2017 (Sweden, Norway)	Patient Education and Counseling	To compare student nurses’ communication self-efficacy, empathy, and mindfulness across two countries, and to analyze the relationship between these qualities	A quantitative design with a questionnaire	156 student nurses from two universities	Langer Mindfulness Scale (α = .839)	Socio-cognitive mindfulness and communication self-efficacy (r = .467, p < .001); socio-cognitive mindfulness and empathy (r = .494, p < .001). Socio-cognitive mindfulness to communication self-efficacy (β = .526, p = .012)	Student nurses scoring high on socio-cognitive mindfulness rated their communication self-efficacy higher than students scoring low on mindfulness. Socio-cognitive mindful learning could improve communication self-efficacy and may consequently have a positive effect on communication skills training.
*Rush, 2018 (USA)	Unpublished master’s thesis	To evaluate the helpfulness of a four-week mindfulness program in psychiatric nurses	A mixed method study with both quantitative and qualitative data	22 nurses in three psychiatric units of a hospital	*Intervention program (4 weeks) Week 1: Discuss stress and burnout consequences and learn Langer’s approaches to mindfulness vs. mindlessness. Weeks 2 and 3: Practice four key features of socio-cognitive mindfulness, focusing on novelty seeking and engagement in Week 2 and focusing on novelty producing and flexibility in Week 3. Week 4: Apply mindfulness in the workplace setting for creative solutions to challenges.		The program content was helpful for increasing curiosity, paying attention to small details, using multiple perspectives, and engaging in mindfulness practice. The majority in the focus group expressed a desire to integrate mindfulness into their practice and believed that mindfulness could assist with management of stress and burnout; they agreed that the program increased their knowledge and capability to practice mindfulness.
Lee & Jang, 2021a (South Korea)	Nurse Educator	To examine the relationships between meditative and socio-cognitive mindfulness and between mindfulness, achievement emotions, and academic outcomes in nursing students	A quantitative design with a questionnaire	459 nursing students from three universities	Langer Mindfulness Scale (α = .89; .73, .86, .69, and .71 for novelty seeking, novelty producing, flexibility, and engagement, respectively)	Socio-cognitive mindfulness and positive achievement emotions (r = .55, p < .001); socio-cognitive mindfulness and negative achievement emotions (r = −.43, p < .001); socio-cognitive mindfulness and academic outcomes (r = .22, p < .001)	Socio-cognitive mindfulness is related to enhancing students’ positive emotions and reducing negative emotions, ultimately influencing their academic outcomes. Mediating effects of achievement emotions between socio-cognitive mindfulness and academic outcomes emphasize the benefits of positive emotions and disadvantages of negative emotions between mindfulness and outcomes.
Lee & Jang, 2021b (South Korea)	Healthcare	To investigate relationships between nursing students’ socio-cognitive mindfulness, emotion regulation, and achievement emotions, and to explore the mediating effects of emotion regulation	A quantitative design with a questionnaire	459 nursing students from three universities	Langer Mindfulness Scale (α = .89; .73, .86, .69, and .71 for novelty seeking, novelty producing, flexibility, and engagement, respectively)	Socio-cognitive mindfulness positively influenced reappraisal: novelty seeking (β = .389, p < .001), novelty producing (β = .248, p < .01), flexibility (β = .398, p < .001), and engagement (β = .225, p < .01); socio-cognitive mindfulness negatively influenced suppression; engagement (β = −.329, p < .001). Socio-cognitive mindfulness positively influenced positive achievement emotions: novelty seeking (β = .516, p < .001), novelty producing (β = .469, p < .001), flexibility (β = .508, p < .001), and engagement (β = .457, p < .001); socio-cognitive mindfulness negatively influenced negative emotions: novelty seeking (β = −.387, p < .001), novelty producing (β = −.315, p < .001), flexibility (β = −.310, p < .001), and engagement (β = −.490, p < .001) Reappraisal mediated the link between socio-cognitive mindfulness and positive emotions: between novelty seeking and positive emotions (a X b = .085, p < .01); between novelty producing and positive emotions (a X b = .052, p < .01); between flexibility and positive emotions (a X b = .091, p < .01); between engagement and positive emotions (a X b = .053, p < .01). Suppression mediated the link between socio-cognitive mindfulness and negative emotions; between engagement and negative emotions (a X b = –.064, p < .01).	Socio-cognitive mindfulness may be effective in regulating emotions among nursing students by enhancing reappraisal and reducing suppression. Socio-cognitive mindfulness also allows nursing students to experience more positive emotions and less negative emotions. Mediating effects of emotion regulation in the relationship between socio-cognitive mindfulness and achievement emotions highlight the relevance of students’ emotion regulation in nursing education, suggesting the need to develop emotion regulation education programs.
Lee, 2022 (South Korea)	International Journal of Environment-al Research and Public Health	To examine the relationships between grit, socio-cognitive mindfulness, and achievement emotions among nursing students, as well as the mediating effects of socio-cognitive mindfulness	A quantitative design with a questionnaire	220 nursing students in a university	Langer Mindfulness Scale (α = .88; .73, .80, .60, and .69 for novelty seeking, novelty producing, flexibility, and engagement, respectively)	Grit and socio-cognitive mindfulness (r = .310, p < .01); grit and positive achievement emotions (r = .433, p < .01); socio-cognitive mindfulness and positive achievement emotions (r = .607, p < .01); grit and negative achievement emotions (r = −.465, p < .01); socio-cognitive mindfulness and negative emotions (r = −.370, p < .01). Grit to socio-cognitive mindfulness (β = .310, p < .01); grit to positive emotions (β = .271, p < .01); grit to negative emotions (β = −.388, p < .01); socio-cognitive mindfulness to positive emotions (β = .524, p < .01); socio-cognitive mindfulness to negative emotions (β = −.249, p < .01). Socio-cognitive mindfulness was mediating the link between grit and positive achievement emotions (a × b = 0.162, p < 0.01) as well as the link between grit and negative achievement emotions (a × b = −.077, p < .01) in nursing students.	The grittier students might have become more mindful in the nursing education context, and they tended to have a higher level of positive emotions and a lower level of negative emotions. Individuals equipped with a higher level of socio-cognitive mindfulness pay more attention to the present moment and thereby improve cognitive flexibility and insights, which facilitates students’ flexible thinking, learning skills, and cognitive outcomes. This would ultimately promote students’ positive emotions and reduce negative emotions. The mediating role of socio-cognitive mindfulness highlights the importance of socio-cognitive mindfulness in the context of nursing education.
Lee & Park, 2022 (South Korea)	BMC Nursing	To examine the relationships between socio-cognitive mindfulness, emotion regulation, and empathy among nurses as well as the mediating effects of emotion regulation	A quantitative design with a questionnaire	245 clinical nurses from two university hospitals	Langer Mindfulness Scale (α = .89; .72, .83, .62, and .69 for novelty seeking, novelty producing, flexibility, and engagement, respectively)	Socio-cognitive mindfulness and reappraisal (r = .375, *p* < .01); socio-cognitive mindfulness and suppression (r = −.147, p < .05); socio-cognitive mindfulness and empathy (r = .584, *p* < .01); reappraisal and empathy (r = .444, *p* < .01); suppression and empathy (r = −.125, *p* < .05). Socio-cognitive mindfulness to reappraisal (β = .404, p < .01); socio-cognitive mindfulness to suppression (β = −.149, p < .05); socio-cognitive mindfulness to empathy (β = .402, p < .01); reappraisal to empathy (β = .341, p < .01); suppression to empathy (β = −.127, p < .05). Emotion regulation of reappraisal mediated the association between socio-cognitive mindfulness and empathy (a X b = .107, p < .01).	The study confirmed significant relationships between socio-cognitive mindfulness, emotion regulation, and empathy among nurses. The mediating role of emotion regulation highlights the relevance of emotion regulation to enhance empathy in the nursing context. The findings indicate that socio-cognitive mindfulness is effective in improving empathy among nurses by enhancing reappraisal.

Note:

*Intervention study.

### Effects of socio-cognitive mindfulness on nurses (Research question 1)

Two studies quantitatively investigated the effects of socio-cognitive mindfulness on nurses. They mainly reported the effectiveness of socio-cognitive mindfulness on nurses’ burnout, emotion regulation, and empathy. Table 1 displays the detailed results of the studies.

First, Heard [42] conducted a cross-sectional study to determine the relationship of socio-cognitive mindfulness with comfort, work satisfaction, and burnout (i.e., emotional exhaustion and depersonalization) in nurses. The main instrument, the LMS [47] which assesses the level of socio-cognitive mindfulness, includes four key features of socio-cognitive mindfulness: novelty seeking, novelty producing, flexibility, and engagement. The results showed that socio-cognitive mindfulness was negatively related to burnout, specifically to emotional exhaustion and depersonalization. In addition, socio-cognitive mindfulness positively influenced personal accomplishment.

Second, Lee and Park [44] examined the relationships between socio-cognitive mindfulness, emotion regulation, and empathy among nurses. They found that socio-cognitive mindfulness had a positive influence on emotion regulation of reappraisal but a negative influence on emotion regulation of suppression, and that socio-cognitive mindfulness had a positive effect on empathy. Regarding the relationships between the emotion regulation strategies and empathy, reappraisal had a positive influence on empathy, but suppression had a negative influence on empathy.

### Effects of socio-cognitive mindfulness on nursing students (Research question 2)

Four studies examined the effects of socio-cognitive mindfulness on nursing students. Their main findings included the relationship of socio-cognitive mindfulness with nursing students’ communication self-efficacy, emotion regulation, achievement emotions, and academic outcomes. The detailed results of the studies are described in Table 1.

First, Sundling et al. [45] investigated the relationships between socio-cognitive mindfulness, communication self-efficacy, and empathy using cross-sectional data. They found that nursing students’ socio-cognitive mindfulness was positively associated with communication self-efficacy and empathy. In addition, socio-cognitive mindfulness positively predicted communication self-efficacy.

Second, Lee and Jang’s [40] study examined the relationships between meditative and socio-cognitive mindfulness and between mindfulness, achievement emotions, and academic outcomes in nursing students. The findings related to socio-cognitive mindfulness showed that socio-cognitive mindfulness was positively associated with positive achievement emotions (e.g., enjoyment, hope, and pride) and academic outcomes, but it was negatively associated with negative achievement emotions (e.g., boredom, anger, anxiety, hopelessness, and shame).

Third, Lee and Jang’s [41] later study examined the relationships between socio-cognitive mindfulness, emotion regulation, and achievement emotions in nursing students. The findings showed that nursing students’ socio-cognitive mindfulness had a positive effect on the adaptive emotion regulation strategy (i.e., reappraisal); specifically, socio-cognitive mindfulness’s four sub-categories of novelty seeking, novelty producing, flexibility, and engagement positively influenced reappraisal. However, socio-cognitive mindfulness had a negative effect on the maladaptive emotion regulation strategy (i.e., suppression); for example, sub-category of engagement negatively influenced suppression. Furthermore, nursing students’ socio-cognitive mindfulness positively predicted positive achievement emotions: novelty seeking, novelty producing, flexibility, and engagement. In contrast, socio-cognitive mindfulness negatively predicted negative achievement emotions: novelty seeking, novelty producing, flexibility, and engagement.

Finally, Lee’s [43] recent study investigated the relationships between grit, socio-cognitive mindfulness, and achievement emotions in nursing students. In particular, this study explored the mediating effects of socio-cognitive mindfulness in the relationship between grit and achievement emotions. The result showed that grit was positively associated with socio-cognitive mindfulness and positive achievement emotions, but negatively associated with negative emotions; socio-cognitive mindfulness correlated positively with positive emotions but correlated negatively with negative emotions. Importantly, socio-cognitive mindfulness was mediating the link between grit and positive achievement emotions as well as the link between grit and negative achievement emotions in nursing students.

### Interventions applying socio-cognitive mindfulness and their effectiveness in nursing practice and nursing education (Research question 3)

Among seven studies, only one [46] conducted an intervention study applying socio-cognitive mindfulness and identifying its effects in the nursing field. Rush [46] analyzed qualitative data of group discussion to evaluate the effect of a socio-cognitive mindfulness intervention program among nurses. The program was conducted for four weeks with 22 nurses in psychiatric units. In week 1, the participants discussed stress and burnout consequences and learned Langer’s approaches to mindfulness vs. mindlessness. In weeks 2 and 3, they practiced four key features of socio-cognitive mindfulness, focusing on novelty seeking (ways to prevent mindlessness) and engagement (noticing details or changes in the environment) in week 2 as well as novelty producing (processing stimuli to create new categories or information) and flexibility (considering multiple perspectives and using the environment as feedback) in week 3. In week 4, they applied mindfulness in the workplace setting for creative solutions to challenges.

In the focus group discussion, the majority expressed a desire to integrate mindfulness into their practice and believed that mindfulness could assist with management of stress and burnout. The participants mentioned that they would employ socio-cognitive mindfulness in their workplace as well as in their daily life, with the majority of the nurses (87.5%) agreeing with the usefulness of the socio-cognitive mindfulness program. Specifically, they found the program content effective in enhancing curiosity, attention to details, adopting various perspectives, and engaging in mindfulness. One participant shared practicing mindfulness during quiet moments either early in the morning or late at night, minimizing distractions for a more focused experience. The participants did not express any significant barriers to attend the program. Overall, the socio-cognitive mindfulness intervention program proved effective in practicing mindfulness techniques and integrating them to the nursing context.

## Discussion

This scoping review aimed to identify the effects of socio-cognitive mindfulness on nurses and nursing students, as well as socio-cognitive mindfulness interventions and their effectiveness in nursing practice and nursing education. The review adopted the Arksey and O’Malley [34] framework following six stages. An electronic search of PubMed, EMBASE, Cochrane Library, CINAHL, Web of Science, ERIC, and ProQuest databases was conducted. The findings of the scarce existing studies have revealed several benefits of applying socio-cognitive mindfulness to the nursing practice and nursing education contexts.

First of all, regarding the effects of socio-cognitive mindfulness on nurses (Research question 1), nurses with a higher level of socio-cognitive mindfulness experienced higher levels of personal accomplishment and a lower rate of burnout or emotional exhaustion [42]. In addition, nurses with higher socio-cognitive mindfulness used the more effective emotion regulation strategy of reappraisal and possessed a higher level of empathy [44]. Heard [42] emphasized that nurses’ flexibility and engagement with increased self-awareness and genuine involvement with patients, main characteristics of socio-cognitive mindfulness, may have enhanced their personal accomplishment but reduced emotional exhaustion and depersonalization. Specifically, more mindful nurses would have fewer problems with the experience of depersonalization and emotional exhaustion; they would seem to experience higher levels of personal accomplishment, thus possibly making them less prone to experience burnout in their work.

Socio-cognitive mindfulness characterized by concentration on the present, openness, and cognitive flexibility will help nurses view nursing environments or situations from multiple viewpoints by considering contexts, eventually producing useful and creative alternatives. Therefore, nurses with socio-cognitive mindfulness could reduce stress perception and burnout while performing nursing practice, as reflected in Heard’s [42] study. In addition, the socio-cognitive mindful process of discovering new distinctions from work environments would help nurses enhance their attention to contexts and retain more open attitudes. This also enables nurses to think about situations from multiple perspectives, helping them regulate their emotions more effectively [44]. For example, when nurses are engaged in active observation of nursing environments that include patients, colleagues, events, or situations, they will have more opportunities to change the way in which they think about disadvantageous situations. This is associated with reappraisal, which is a useful strategy in regulating emotions. Reappraisal can improve nurses’ emotional status by generating more positive feelings, ultimately improving nursing practice for patients [44, 48].

Second, nursing students with a higher level of socio-cognitive mindfulness possessed higher communication self-efficacy compared to the students with lower socio-cognitive mindfulness [45] (Research question 2). This finding supports that mindful learning could improve nursing students’ communication self-efficacy and possibly have a positive influence on communication skills training. In Lee and Jang’ s [40] study, socio-cognitive mindfulness was related to enhancing students’ positive achievement emotions and reducing negative achievement emotions, which could ultimately influence their academic outcomes. In their later investigation, Lee and Jang [41] stated that socio-cognitive mindfulness could encourage students to adopt a reappraisal strategy by accepting new attributes and aspects of information from various perspectives, and guide thoughts to an ideal direction in adverse situations, possibly inhibiting suppression at the same time. Socio-cognitive mindfulness enables students to be flexible and open in their thoughts, so they can expand their cognitive performance and improve learning skills and creativity [9]. By being cognitively flexible, students have the ability to respond creatively to the challenges encountered in learning situations, which could increase their interest in learning [49]. This will encourage students to become more engaged in academic activities, ultimately facilitating positive learning emotions while reducing negative learning emotions, as reflected in the reviewed studies [40, 41, 43].

In addition, Lee’s [43] study claims that socio-cognitive mindfulness might have enhanced nursing students’ positive emotions and diminished their negative emotions, since students with higher socio-cognitive mindfulness would focus more on the present moment and thereby improve insights. This would foster students’ flexible thinking, learning skills, and cognitive outcomes. Notably, the mediating effects of socio-cognitive mindfulness emphasized that socio-cognitive mindfulness might be one mechanism to explain the relationship between nursing students’ grit and achievement emotions. This supports the significance of socio-cognitive mindfulness in nursing education, highlighting the need of practical mindfulness programs to nurture nursing students’ socio-cognitive mindfulness. When students exhibit socio-cognitive mindful attitudes, they can accept new attributes and various aspects of given information after evaluating them in many perspectives [9, 28]. In this process, they can establish a new kind of thinking ability that can convert the way they think even in unfavorable learning situations, meaning that they can use a reappraisal strategy, adaptive emotion regulation [41, 43]. They are able to consider situations in the contexts utilizing multiple viewpoints and change their thoughts in a desirable direction, which promotes a reappraisal strategy but prohibits a suppression strategy [41, 43]. This evidences that socio-cognitive mindfulness can help nursing students enhance their emotional experiences, and that it can be beneficial for them to apply an effective emotion regulation strategy rather than an ineffective one.

Finally, regarding socio-cognitive mindfulness interventions applied in nursing practice and nursing education and their effects (Research question 3), one study by Rush [46] found that the socio-cognitive mindfulness program helped nurses cope with stress and emotional exhaustion more effectively and view the nursing environments from various perspectives. This program was beneficial for nurses to increase their curiosity, pay attention to small details, approach situations with multiple perspectives, and engage in mindfulness practice. In reality, nurses constantly suffer from burnout or emotional exhaustion on a regular basis at work. Mindful nurses could make an effort to view work tasks from various perspectives and engage themselves in tasks more genuinely and actively to create a less stressful workplace, as indicated in Rush [46].

According to Langer and Moldoveanu’s [49] assumptions, nurses could experience burnout because they are interrupted by old categories or old mindsets. Adherence to old mindsets, for example, feeling comfortable with “the way we always do,” might produce burnout along with job dissatisfaction. This is true of most nursing practice environments. Nurses are likely to continue with the ways they have always executed certain interventions, possibly contributing to their burnout. Nevertheless, nurses with higher socio-cognitive mindfulness might have tried to avert burnout by creating diversity in their nursing tasks and including more flexibility [46, 49].

### Implications

Despite the potential benefits of applying socio-cognitive mindfulness to the nursing field discussed so far, surprisingly, nursing researchers have very rarely considered applying interventions based on socio-cognitive mindfulness. Through this scoping review, we strongly recommend conducting intervention studies applying socio-cognitive mindfulness to nursing practice and nursing education. As a start, researchers can include simple and time-saving techniques, such as those introduced by Carson and Langer [26]. For example, nurses can actively observe novel distinctions, potentially promoting positive emotions and interest in objects, events, and situations. This allows for active mental exploration through their mindful and non-judgmental thinking, which can also enhance their self-acceptance by exploring new aspects of themselves [26]. Nurses can also view situations from the different perspectives of other people, which would help them become more mindful and accepting. This mindfulness could be utilized to cultivate decent values and attitudes such as tolerance, generosity, and respect for creating more diversity at the workplace [33].

These kinds of easily applicable strategies are exactly what nurses need to regulate their emotions more effectively so that they can experience more positive emotions and less negative emotions in nursing practice situations. In addition, socio-cognitive mindfulness approach would be feasible for “busy” nurses to implement in nursing practice environments where they struggle with restrictive tasks [46]. It should also be noted that nurses can apply these mindful strategies to their personal lives in addition to their workplace. Through self-awareness and self-acceptance of socio-cognitive mindfulness, nurses will have a chance to look at themselves and care for themselves first, before actually nursing their patients. This is an important point, considering that nurses tend to feel obligated to care for others before themselves; however, nurses are likely to perform better nursing practice once they first become “happy” nurses. Since socio-cognitive mindfulness can be activated as a result of quite simple methods without long-term mediation, this mindfulness could be more adaptable to nurses in hectic health organizations.

As for the nursing education context, researchers can include the following strategies as interventions applying socio-cognitive mindfulness. For instance, nursing students could be assigned to perform activities to approach nursing practicum environments from multiple perspectives and acknowledge a variety of possible alternatives from their peers. They could also consider simulation scenarios in a variety of different contexts and openly discuss various solutions with their peers. This would help them enhance critical thinking, in-depth understanding, and decision making, which are crucial for effective learning. They would also become more mindful and embracing, which could improve their college life in general. As another strategy, they could keep a “mindfulness” journal. They could invest a little time to reflect on their daily life, including their learning status. This habit would definitely help them grow more mindful, guiding their life to a better orientation.

### Limitations

Notwithstanding the significant contribution to the nursing literature in that this review is the first study to provide an overview of the existing studies on socio-cognitive mindfulness in nursing, there are limitations to consider. First, although we carried out a systematic search strategy, studies on socio-cognitive mindfulness in the nursing field in other languages might have been excluded, since the search was limited to papers in the English language only. Second, four studies among the seven included in this review originated from South Korea that included the same author. This might have resulted in biased discussions related to socio-cognitive mindfulness in this review. In particular, two theses were included in the seven studies, and they may have weak methodological quality due to the absence of rigorous peer-review processes. Finally, although we initially aimed to include papers from a broad range of countries, only seven studies from United States, South Korea, Sweden, and Norway were included in the final analysis; there exists a geographic limitation which might restrict the ability to generalize the conclusions.

While the reviewed studies provide valuable insights into the role of socio-cognitive mindfulness within specific cultural contexts, caution is needed when applying the findings to a broader and more diverse population regarding the relationship with nurses’ psychological well-being. Scoping reviews, unlike systematic reviews, aim to present an overview of the existing evidence base without rigorously evaluating quality; thus, no formal assessment is generally conducted on the methodological quality of the included studies [50]. The present study also did not conduct a quality assessment of the included studies. Nevertheless, the present study has provided an overall map of the literature on the overlooked research topic of socio-cognitive mindfulness in nursing to inform researchers and call for further investigation, which is another important purpose of the scoping review method.

## Conclusion

This scoping review addresses the concept of Langer’s socio-cognitive mindfulness, which has recently started gaining nursing researchers’ attention. This study introduces socio-cognitive mindfulness and suggests applying it to the nursing field. To this end, this research reviewed existing studies to identify what is known in the research field of socio-cognitive mindfulness in the nursing field. Based on the present review findings, we discussed the significance of socio-cognitive mindfulness in nursing practice and nursing education. Additionally, we suggested some feasible strategies of socio-cognitive mindfulness that could be applied to the nursing field as interventions.

In conclusion, integrating socio-cognitive mindfulness into nursing practice and education is crucial for enhancing the well-being and effectiveness of healthcare professionals. Socio-cognitive mindfulness can play an important role in promoting nurses’ positive emotions and effective emotion regulation, improving their psychological well-being, which ultimately will have a positive influence on their nursing performance for their patients. For example, by actively observing novel distinctions in their surroundings and adopting different perspectives, nurses can cultivate socio-cognitive mindfulness. Socio-cognitive mindfulness will also be advantageous for nursing students in that they will be able to experience more positive learning emotions and fewer negative ones. As well, they will be able to more efficiently manage their emotions, which will improve academic outcomes and the quality of college life. To become more mindful, nursing students can approach nursing practicum environments from various perspectives and consider different solutions in simulation scenarios. They could also maintain a mindfulness journal, allocating a brief time for daily reflection.

We hope that this contribution will raise nursing researchers’ awareness of the significance of socio-cognitive mindfulness in nursing, and encourage them to actively conduct further research on this under-examined area in nursing. Furthermore, based on more empirical evidence in the future, it is advised to develop a program so that nurses and nursing students can acquire socio-cognitive mindfulness, apply it to the nursing field, and evaluate its effects.

## Supporting information

S1 File Search strategy. (DOCX)

S1 Checklist PRISMA checklist. (DOCX)
