# Correction: Socio-cognitive mindfulness in nursing: A scoping review

**DOI:** 10.1371/journal.pone.0303616

**Published:** 2024-05-09

**Authors:** The PLOS ONE Staff

The published funding statement is incorrect. The publisher apologizes for this error.

The correct funding statement is as follows: This research received no specific grant from any funding agency in the public, commercial, or not-for-profit sectors.
