# Supplementary material for: Socio-cognitive mindfulness in nursing: A scoping review
Source: PLoS One. 2024 Apr 29;19(4):e0300459. doi: 10.1371/journal.pone.0300459 (PMC11057744; doi:10.1371/journal.pone.0300459)
Supplement: S1 File — (DOCX) [file pone.0300459.s001.docx]

**Search strategy**

| No. | Query | Results |
| --- | --- | --- |
| #1 | nurse[MeSH Terms] | 97,254 |
| #2 | nursing[MeSH Terms] | 262,552 |
| #3 | Students, Nursing[MeSH Terms] | 29,802 |
| #4 | mindfulness[MeSH Terms] | 6,058 |
| #5 | nurs*[Title/Abstract] | 523,551 |
| #6 | mindfulness[Title/Abstract] | 12,085 |
| #7 | (((nurse[MeSH Terms]) OR (nursing[MeSH Terms])) OR (Students, Nursing[MeSH Terms])) OR (nurs*[Title/Abstract]) | 677,372 |
| #8 | (mindfulness[MeSH Terms]) OR (mindfulness[Title/Abstract]) | 12,746 |
| #9 | ((((nurse[MeSH Terms]) OR (nursing[MeSH Terms])) OR (Students, Nursing[MeSH Terms])) OR (nurs*[Title/Abstract])) AND ((mindfulness[MeSH Terms]) OR (mindfulness[Title/Abstract])) | 834 |

**DATABASE #1.**

PubMed MELINE on February 09, 2023

**DATABASE #2.**

Embase on February 09, 2023

| No. | Query | Results |
| --- | --- | --- |
| #1 | 'nursing'/exp | 419,893 |
| #2 | 'nurse'/exp | 213,361 |
| #3 | 'nursing student'/exp | 32,301 |
| #4 | 'mindfulness'/exp | 14,267 |
| #5 | nurs*:ti,ab,kw | 628,865 |
| #6 | mindfulness:ti,ab,kw | 15,392 |
| #7 | 'nursing'/exp OR 'nurse'/exp OR 'nursing student'/exp OR nurs*:ti,ab,kw | 862,655 |
| #8 | 'mindfulness'/exp OR mindfulness:ti,ab,kw | 18,410 |
| #9 | ('nursing'/exp OR 'nurse'/exp OR 'nursing student'/exp OR nurs*:ti,ab,kw) AND ('mindfulness'/exp OR mindfulness:ti,ab,kw) | 1,160 |

**DATABASE #3.**

Cochrane Library on February 09, 2023

| No. | Query | Results |
| --- | --- | --- |
| #1 | MeSH descriptor: [Nursing] explode all trees | 4,055 |
| #2 | MeSH descriptor: [Nurses] explode all trees | 1,583 |
| #3 | MeSH descriptor: [Students, Nursing] explode all trees | 694 |
| #4 | (nurs*):ti,ab,kw (Word variations have been searched) | 51,900 |
| #5 | MeSH descriptor: [Mindfulness] explode all trees | 1,675 |
| #6 | (mindfulness):ti,ab,kw (Word variations have been searched) | 12,696 |
| #7 | #1 or #2 or #3 or #4 | 52,211 |
| #8 | #5 or #6 | 12,706 |
| #9 | #7 AND #8 | 613 |

**DATABASE #4.**

CINAHL on February 09, 2023

| No. | Query | Results |
| --- | --- | --- |
| S1 | (MH "Nurses+") | 239,164 |
| S2 | (MH "Students, Nursing+") | 44,401 |
| S3 | TI nurs* OR AB nurs* | 622,995 |
| S4 | (MH "Mindfulness+") | 7,682 |
| S5 | TI mindfulness OR AB mindfulness | 8,469 |
| S6 | S1 OR S2 OR S3 | 720,902 |
| S7 | S4 OR S5 | 11,064 |
| S8 | S6 AND S7 | 1,133 |

**DATABASE #5.**

Web of Science on February 09, 2023

| No. | Search Query | Results |
| --- | --- | --- |
| #1 | TS=(nurs*) | 364,884 |
| #2 | TS=(mindfulness) | 24,469 |
| #3 | #1 AND #2 | 1,109 |

**DATABASE #6.**

ERIC (EBSCO) on February 09, 2023

| No. | Query | Results |
| --- | --- | --- |
| S1 | DE "Nursing" | 2,209 |
| S2 | DE "Nursing Students" | 1,357 |
| S3 | DE "Nurses" | 3,367 |
| S4 | DE "Metacognition" | 11,902 |
| S5 | TI nurs* OR AB nurs* | 16,899 |
| S6 | TI mindfulness* OR AB mindfulness* | 1,455 |
| S7 | S1 OR S2 OR S3 OR S5 | 17,396 |
| S8 | S4 OR S6 | 12,331 |
| S9 | S7 AND S8 | 74 |

**DATABASE #7.**

ProQuest on February 09, 2023

| No. | Query | Results |
| --- | --- | --- |
| S1 | MAINSUBJECT.EXACT("Nursing") | 243,379 |
| S3 | MAINSUBJECT.EXACT("Nurses") | 350,918 |
| S4 | MAINSUBJECT.EXACT("Mindfulness") | 42,000 |
| S5 | title(mindfulness) OR abstract(mindfulness) | 23,003 |
| S6 | title(nurs*) OR abstract(nurs*) | 1,044,446 |
| S7 | MAINSUBJECT.EXACT("Nursing") OR MAINSUBJECT.EXACT("Nurses") OR (title(nurs*) OR abstract(nurs*)) | 1,303,273 |
| S8 | MAINSUBJECT.EXACT("Mindfulness") OR (title(mindfulness) OR abstract(mindfulness)) | 55,911 |
| S9 | (MAINSUBJECT.EXACT("Nursing") OR MAINSUBJECT.EXACT("Nurses") OR (title(nurs*) OR abstract(nurs*))) AND (MAINSUBJECT.EXACT("Mindfulness") OR (title(mindfulness) OR abstract(mindfulness))) | 875 |
